# Infant and young child feeding in rural Bangladesh: insights into knowledge, practices, and associated factors

**DOI:** 10.1017/jns.2026.10121

**Published:** 2026-06-29

**Authors:** Zihadul Sheikh, Md. Shahadoth Hossain, Masum Ali, Rafid Hassan, Md. Mahbub Alam, Md Ruhul Amin

**Affiliations:** 1 Institute of Nutrition and Food Science, University of Dhaka, Dhaka, Bangladesh; 2 Department of Nutrition and Food Engineering, Daffodil International University, Bangladesh; 3 Nutrition and Health Science, Laney Graduate School, Emory University, USA; 4 Nutrition Research Division, ICDDRB, Bangladesh

**Keywords:** Breastfeeding, Complementary feeding, Infant feeding, Nutrition knowledge and practice, Rural Bangladesh, Young child feeding

## Abstract

Optimal infant and young child feeding (IYCF) practices are essential for ensuring child growth, development, and survival. However, comprehensive evidence on IYCF knowledge, practices, and their determinants among rural mothers in Bangladesh remains limited. This study aimed to assess IYCF knowledge and practices and to identify the factors associated with these outcomes among rural mothers in Bangladesh. This study utilised data from the third round of the Bangladesh Integrated Household Survey (BIHS), focusing on rural mothers aged 15–49 years with children aged 0–23 months. A total of 873 samples were included in the study. Descriptive statistics and logistic regression analyses were utilised to investigate the prevalence and determinants of IYCF knowledge and practices. Findings revealed that only 30.3% of mothers had adequate IYCF knowledge, and an equal proportion adhered to overall IYCF practices. Multivariate regression analysis showed that maternal education, geographic region, and household wealth quintile were significant predictors of IYCF knowledge. Breastfeeding practices were associated with the child’s age, place of delivery, geographic region, household wealth, and maternal IYCF knowledge. Complementary feeding practices were mainly influenced by the child’s age, sex, and household wealth quintile. Overall adherence to recommended IYCF practices was significantly associated with the child’s age and geographic region. This study reveals persistently low IYCF knowledge and overall IYCF practices among rural mothers in Bangladesh. Strengthening maternal education, promoting community-based nutrition counselling, expanding access to health services in underserved regions, and targeting low-income households with tailored support are essential to improve child feeding practices.

## Graphical abstract

**Figure:**
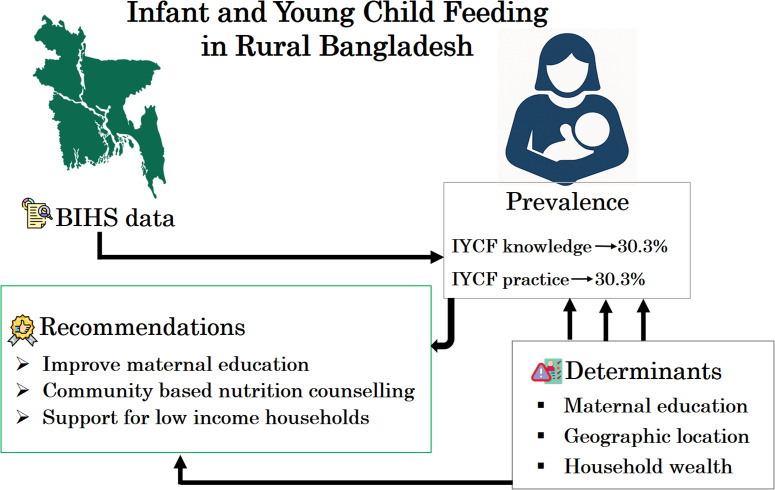

## Introduction

Optimal infant and young child feeding (IYCF) practices are critical for preventing malnutrition and promoting healthy growth in early childhood. Malnutrition remains a major public health concern among children in low- and middle-income countries, contributing to approximately 45% of deaths among children under five years of age globally.^(1)^ Optimal breastfeeding can prevent 13% of deaths in children under five years old, while proper complementary feeding can further reduce these deaths by an additional 6%.^(2)^ Adequate breastfeeding for all children aged 0–23 months could save almost 820,000 lives annually for children under the age of 5.^(1)^ The development, growth, and potential of a child depend heavily on early childhood feeding practices. The best period to establish lifelong dietary habits is in infancy.^(3)^ The first 1,000 days of life from conception to two years of age, are particularly vital, laying the foundation for lifelong health and neurodevelopment.^(4,5)^ For achieving and preserving optimum nutrition and health, appropriate evidence-based feeding practices are crucial.^(6)^

Optimal IYCF practices comprising early initiation of breastfeeding (EIBF) within one hour of birth, exclusive breastfeeding for the first six months, and timely introduction of safe, nutritionally adequate complementary foods – are essential for achieving and maintaining child health.^(1)^ However, despite this, global adherence remains suboptimal. Only 49% of newborns are breastfed within the first hour, and just 44% are exclusively breastfed for six months.^(7)^ Complementary feeding is often inadequate, with less than a quarter of infants aged 6–23 months meeting dietary diversity and feeding frequency recommendations.^(1)^ These challenges are exacerbated by the aggressive marketing of processed foods and widespread misinformation, especially in low-resource settings.^(7)^

Despite economic growth and national nutrition programmes, undernutrition remains a major challenge in Bangladesh, with 22% of children under five underweight, 24% stunted, and 11% wasted.^(8)^ Exclusive breastfeeding rates stand at 55%, while minimum dietary diversity, meal frequency, and acceptable diet rates are 39%, 61%, and 29%, respectively.^(8)^ Poor IYCF practices contribute significantly to undernutrition, particularly in rural areas where both feeding practices and child nutrition are worse than in urban settings.^(9)^ Rural mothers often face additional barriers such as limited access to nutrition information, lower educational attainment, and restricted exposure to health and media messages, which may hinder adequate knowledge and optimal IYCF practices.^(9–11)^ Cultural beliefs and limited utilisation of maternal health services may further influence feeding behaviours in rural communities.^(12,13)^ Evidence from rural Bangladesh indicates notable gaps in complementary feeding knowledge and practices; complementary feeding initiation was early for 7%, timely for 49%, and late for 44% of infants, while only 19% of mothers knew the WHO-recommended age for initiation^.(14)^ Similarly, only about half of mothers reported correct knowledge of recommended complementary feeding practices, including initiation at six months, appropriate food consistency, quantity, and quality.^(15)^

Evidence from earlier research indicates that improved IYCF knowledge positively influences practices and child nutritional outcomes, including reductions in stunting and wasting.^(16,17)^ Maternal knowledge is influenced by factors such as age, education, place of delivery, paternal education and support, prior exposure to IYCF information, spousal discussion, and antenatal care visits,^(18)^ while maternal education, health service use, autonomy, husband’s occupation, media access, and child’s sex influence IYCF practices.^(19,20)^ Understanding these determinants is critical for designing effective strategies to improve maternal knowledge and child feeding practices, particularly in rural communities where access to information and services may be limited.

While large-scale surveys such as the Bangladesh Demographic and Health Survey (BDHS) and the Multiple Indicator Cluster Survey (MICS) provide national estimates of IYCF practices, they do not capture maternal IYCF knowledge. Existing district-level studies in areas such as Dhaka, Noakhali, Cox’s Bazar, Jamalpur, Jashore Sylhet, and Moulvibazar are limited in scope, lack national representativeness, and often assess either knowledge or practices, but rarely both.^(11,21–28)^ This highlights the need for a dataset, such as Bangladesh Integrated Household Survey (BIHS), that captures both maternal knowledge and IYCF practices within a nationally representative rural population. To address these gaps, this study aimed to assess both IYCF knowledge and practices covering breastfeeding and complementary feeding and to explore their associated factors among rural mothers in Bangladesh using nationally representative household survey data.

## Methods

### Data source

This study was a secondary analysis of data from the BIHS, conducted by the International Food Policy Research Institute (IFPRI) between November 2018 and April 2019 in rural Bangladesh.^(29)^ The survey was funded by the United States Agency for International Development (USAID) and supported in data collection by Data Analysis and Technical Assistance (DATA), Dhaka.

BIHS employed a stratified, two-stage sampling design, covering 325 primary sampling units and 5,604 rural households, and was designed to be nationally representative of rural Bangladesh. Data were collected via structured, face-to-face interviews using a standardised questionnaire. The dataset is publicly accessible through the IFPRI Dataverse, part of the Harvard Data verse network.^(30)^

### Study population

**Figure 1.**
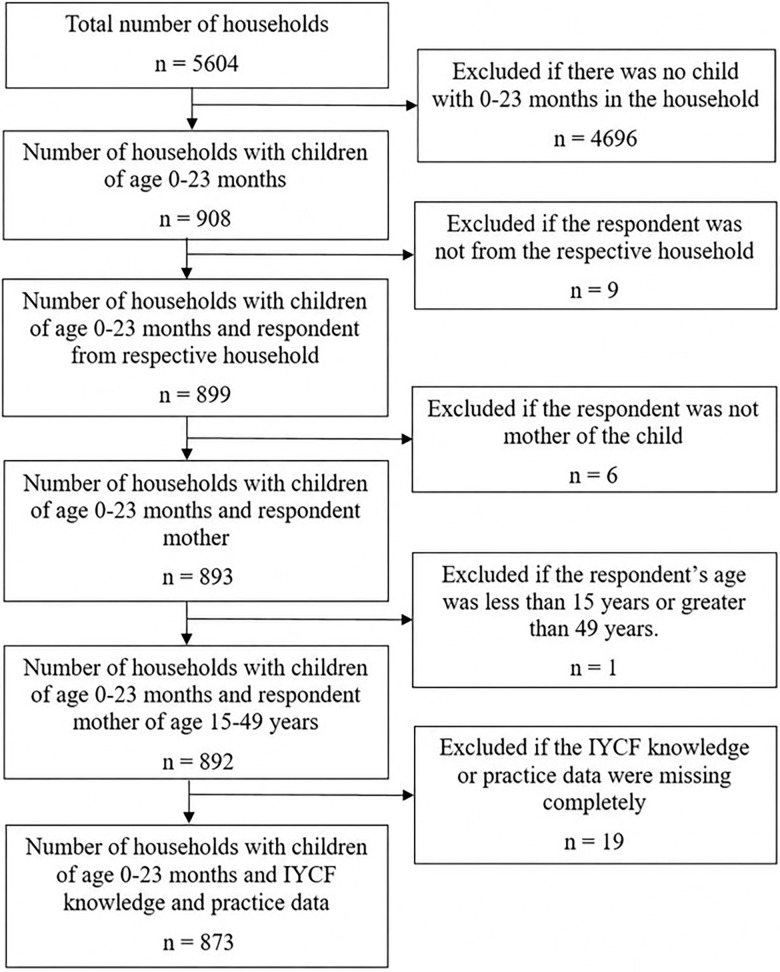
Selection of study participants included in the analysis.

The analysis focused on mothers aged 15–49 years with children aged 0–23 months. Households were excluded if the mother was under 15 years, not identified as a household member, or if data on key IYCF variables were missing. Among 908 eligible households, 9 were excluded for non-household status, 6 for not being mothers, 1 for maternal age below 15 years, and 19 for missing data, resulting in a final sample of 873 households. In households with more than one eligible child, data for only the first child listed in the response order were included. A flow diagram detailing the inclusion process is provided in Figure 1.

### Assessment of IYCF knowledge and practices

The IYCF knowledge of rural mothers was assessed using 18 questions, including 12 single-response and 6 multiple-response items. Responses were coded as ‘Knows’ for correct answers and ‘Does not know’ for incorrect or no answers. Answer accuracy was determined based on WHO and UNICEF recommendations for IYCF practices, while the overall nutrition knowledge assessment followed FAO guidelines.^(31)^ For multiple-response items, participants were classified as ‘Knows’ if they provided at least one correct answer. Each correct response was awarded one point, and total scores were converted to percentages. Knowledge levels were categorised as poor (0–50%), fair (51–80%), and good (>80%). ^(32)^ For regression analysis, knowledge was dichotomised into good (>80%) and inadequate (≤80%).

IYCF practices were assessed using WHO and UNICEF indicators for breastfeeding and complementary feeding.^(33)^ The indicator ‘exclusively breastfed for the first two days after birth (EBF2D)’ was excluded due to data unavailability. Correct practices were scored as one point each, and cumulative scores were calculated. A total score >80% was classified as appropriate practice, while scores ≤80% were considered inappropriate. Practices were evaluated in three domains: overall IYCF practices, breastfeeding practices, and complementary feeding practices. Composite scores for each domain were constructed using relevant WHO/UNICEF indicators.

### Study variables

Independent variables included maternal age, age at first marriage, age at first birth, education level, occupation, religion, child’s age and sex, household wealth quintile, and administrative division. Outcome variables included IYCF knowledge and IYCF practices. IYCF knowledge was treated as both an outcome (in the knowledge model) and an independent variable (in practice models). Although Bangladesh currently has eight administrative divisions, the BIHS dataset included seven, with Mymensingh grouped under Dhaka.

### Statistical analysis

Data were analysed using SPSS version 26 and Microsoft Excel 2019, following a three-step approach. First, descriptive statistics were used to summarise participant characteristics. Categorical variables were presented as frequencies and percentages, and continuous variables as means with standard deviations. Second, bivariate analyses using chi-square tests were conducted to assess associations between independent and dependent variables. Variables with *p*-values ≤ 0.25 in the bivariate analysis were tested for multicollinearity using the variance inflation factor (VIF), with a cut-off of 2.^(34)^ Finally, binary logistic regression was performed to identify factors independently associated with IYCF knowledge and practices. Results were reported as adjusted odds ratios (AORs) with 95% CIs. Statistical significance was defined as a *p*-value < 0.05. Additionally, the unadjusted models, model fit assumptions, and VIF values for the adjusted models are presented in supplementary Tables 1–6. All analyses were adjusted for clustering, stratification, and sampling weights to account for the complex survey design.

### Ethical considerations

BIHS obtained ethical approval from the Institutional Review Board of the International Food Policy Research Institute, Washington, DC, USA (Ref: 2014-44-PHND-D). The Ministry of Agriculture of the Government of Bangladesh reviewed the survey instruments and granted approval to conduct the study. All participants gave their informed verbal consent for participation in the study.

## Results

### Basic characteristics of the study population

Table 1 shows that the mean age of mothers was 26 years, with the largest proportion aged 25–29 years (29.4%). Over half (53%) married before 18 years, and 56.8% had their first child between 15–19 years. Most mothers (88.4%) could read and write, and 42.5% had incomplete secondary education. The majority were housewives (93.8%) and Muslim (91.4%). The mean age of children was 11.1 months, with a slight male predominance (51.8%). Nearly half of the deliveries (46.5%) occurred in health facilities, while 35.3% took place at home. Participants were distributed across all wealth quintiles, with 27.8% in the poorest category. The highest representation was from Dhaka division (30%), followed by Chattogram (20.5%) and Sylhet (16.9%).

**Table 1.** Basic characteristics of the participants (*n* = 873)

Variables	Frequency (percentage)
***Age of mothers (in years)***	
15–19	124 (14.2)
20–24	253 (29)
25–29	257 (29.4)
30–34	164 (18.8)
35–49	75 (8.6)
Mean (SD)	26 ± 5.9
***Age at first marriage (in years)***	
<18	463 (53)
18–24	391 (44.8)
25–49	19 (2.2)
***Age at first birth of the child (in years)***	
15–19	496 (56.8)
20–24	311 (35.6)
25–49	66 (7.6)
***Literacy of the mother***	
Cannot read or write	35 (4)
Can sign only	66 (7.6)
Can read and write	772 (88.4)
***Education status of the mother***	
No education	85 (9.7)
Primary incomplete	110 (12.6)
Primary completed	151 (17.3)
Secondary incomplete	371 (42.5)
Secondary completed	89 (10.2)
More than secondary	67 (7.7)
***Occupational status***	
Housewife	819 (93.8)
Others	54 (6.2)
***Religion***	
Muslim	797 (91.4)
Others	75 (8.6)
***Age of children (in months)***	
0–5	230 (26.3)
6–8	123 (14.1)
9–11	91 (10.4)
12–17	241 (27.6)
18–23	188 (21.5)
Mean ± SD	11.1 ± 6.8
***Gender of children***	
Male	452 (51.8)
Female	421 (48.2)
***Place of delivery***	
Health facility	406 (46.5)
Home	308 (35.3)
Others	159 (18.2)
***Wealth quintile***	
Poorest	243 (27.8)
Poor	209 (23.9)
Middle	191 (21.9)
Rich	132 (15.1)
Richest	98 (11.2)
***Division***	
Dhaka	262 (30)
Chattogram	179 (20.5)
Barishal	68 (7.8)
Khulna	58 (6.7)
Rajshahi	80 (9.2)
Rangpur	78 (8.9)
Sylhet	147 (16.9)

### Distribution of IYCF knowledge and practices among the participants

Most mothers demonstrated high knowledge on key IYCF topics, including initiation of breastfeeding after birth (95.4%), feeding colostrum (94.7%), duration of exclusive breastfeeding (90.4%), and appropriate feeding during illness (>89%). However, knowledge was low for optimal breastfeeding frequency (12.5%) and the need for extra meals after illness (24.6%). In practice, ever breastfeeding (100%) and continued breastfeeding at 12–23 months (93.7%) were widely adopted, whereas EIBF (59.0%), introduction of solid/semi-solid foods at 6–8 months (65.7%), and exclusive breastfeeding under six months (70.3%) were suboptimal. Dietary indicators showed poor adherence to minimum dietary diversity (30.1%), minimum acceptable diet (25.8%), and egg/flesh food consumption (49.9%), with considerable prevalence of unhealthy food consumption (25.4%) and zero vegetable/fruit intake (44.8%) (Table 2).

**Table 2.** Prevalence of IYCF knowledge and practices among the participants Table 2 long description.

Item no.	Knowledge of mothers regarding IYCF	Knows (%)	IYCF indicators	Prevalence (%)
1	Time of initiation of breastfeeding after birth	95.4	Ever breastfed (EvBF)	100.0
2	Feeding colostrum	94.7	Early initiation of breastfeeding (EIBF)	59.0
3	Times of breastfeeding	12.5	Exclusively breastfeeding under six months (EBF)	70.3
4	What to do when a mother thinks that her baby is not getting enough milk	78.0	Mixed milk feeding under six months (MixMF)	15.7
5	Whether Infants under six months should be fed water if the weather is very hot	48.0	Continued breastfeeding 12–23 months (CBF)	93.7
6	Whether breastfeeding infants under six months should be fed water if mothers are pregnant	65.8	Introduction of solid, semi-solid or soft foods 6–8 months (ISSSF)	65.7
7	Duration of exclusive breastfeeding	90.4	Minimum dietary diversity 6–23 months (MDD)	30.1
8	Age to start giving liquid (including water) other than breastmilk	71.8	Minimum meal frequency 6–23 months (MMF)	64.8
9	Age to start giving foods in addition to breastmilk	69.1	Minimum meal feeding frequency for non-breastfed children 6-23 months (MMFF)	26.8
10	Duration of breastfeeding	67.2	Minimum acceptable diet 6–23 months (MAD)	25.8
11	Consequences of iron deficiency in Children	85.3	Egg and/or flesh food consumption 6–23 months (EFF)	49.9
12	Seasoning is often fortified with iodine	55.6	Sweet beverage consumption 6–23 months (SwB)	10.1
13	Duration of the need for extra meals for children after illness	24.6	Unhealthy food consumption 6–23 months (UFC)	25.4
14	What to do regarding child feeding when a child aged less than 6 months has diarrhoea	89.5	Zero vegetable or fruit consumption 6–23 months (ZVF)	44.8
15	What to do regarding child feeding when a child aged more than 6 months has diarrhoea	96.3	Bottle feeding 0–23 months (BoF)	12.8
16	When to wash hands	100		
17	Ways to encourage young children to eat their food	98.1		
18	Foods needed for a young child (<24 months) to grow and develop their brain	99.8		

About 30% of mothers demonstrated good overall IYCF knowledge and overall IYCF practices, while the majority (∼70%) had inadequate knowledge and practice. The mean knowledge and practice scores were 13.4 ± 1.9 (out of 18) and 6.3 ± 2.2 (out of 15), respectively. No significant association was observed between IYCF knowledge and practices (Table 3).

**Table 3.** Association between IYCF knowledge and practice among participants

Categories	IYCF knowledge %	IYCF practice %	*p*-Value*
Good (>80%)	30.3	30.3	0.158
Inadequate (Fair/Poor, ≤80%)	69.7	69.7	
Overall score (Mean ± SD)	13.4 ± 1.9	6.3 ± 2.2	

*Note:* IYCF knowledge score is based on 18 items, and practice score is based on 15 items, *p-Value from the Pearson chi-square test.

### Determinants of overall IYCF knowledge

Mothers with more than secondary education had significantly higher odds of possessing good IYCF knowledge compared to those with no education (AOR = 2.3; 95% CI: 1.0–5.2). Geographical differences were evident – mothers from Chattogram (AOR = 0.6; 95% CI: 0.4–1.0) and Khulna (AOR = 0.3; 95% CI: 0.1–0.7) were less likely, while those from Rangpur (AOR = 1.8; 95% CI: 1.1–3.2) were more likely, to have good IYCF knowledge compared with Dhaka. Wealth status showed a strong positive association: mothers in the ‘poor’ (AOR = 1.7; 95% CI: 1.1–2.7), ‘rich’ (AOR = 2.0; 95% CI: 1.2–3.5), and ‘richest’ (AOR = 2.8; 95% CI: 1.5–5.1) quintiles had higher odds of good knowledge compared to the poorest group (Table 4).

**Table 4.** Factors associated with IYCF knowledge

Characteristics	IYCF knowledge AOR (95% CI)
***Education status of the mother***	
No education	Ref.
Primary incomplete	1.0 (0.5–2.2)
Primary completed	1.4 (0.7–2.8)
Secondary incomplete	1.6 (0.8–2.9)
Secondary completed	1.4 (0.7–3.0)
More than secondary	2.3 (1.0–5.2)*
***Occupational status of the mother***	
Housewife	Ref.
Others	1.6 (0.9–3.0)
***Religion***	
Muslims	Ref.
Others	1.5 (0.9–2.5)
***Age of the child (in months)***	
0–5	Ref.
6–8	1.4 (0.9–2.3)
9–11	1.2 (0.7–2.2)
12–23	1.0 (0.7–1.5)
***Division***	
Dhaka	Ref.
Chattogram	0.6 (0.4–1.0)*
Barishal	1.4 (0.8–2.5)
Khulna	0.3 (0.1–0.7)*
Rajshahi	0.7 (0.4–1.3)
Rangpur	1.8 (1.1–3.2)*
Sylhet	0.7 (0.4–1.1)
***Wealth quintile***	
Poorest	Ref.
Poor	1.7 (1.1–2.7)*
Middle	1.4 (0.8–2.2)
Rich	2.0 (1.2–3.5)*
Richest	2.8 (1.5–5.1)*

*Note:* *p-Value < 0.05.

### Determinants of breastfeeding practices

Appropriate breastfeeding practices were significantly associated with child age, place of delivery, region, and maternal IYCF knowledge. Children aged 6–23 months had higher odds of receiving appropriate breastfeeding (AOR: 3.2; 95% CI: 2.4–4.5) compared to those aged 0–5 months. Compared to children born in health facilities, those born at home (AOR: 1.7; 95% CI: 1.2–2.4) or other locations (AOR: 2.1; 95% CI: 1.4–3.2) had higher odds of appropriate breastfeeding. Mothers in Sylhet Division had significantly better breastfeeding practices (AOR: 1.7; 95% CI: 1.0–2.7), and good IYCF knowledge was also a significant predictor (AOR: 1.5; 95% CI: 1.1–2.1) (Table 5).

**Table 5.** Factors associated with IYCF practice among the participants

Characteristics	Breastfeeding practice AOR (95% CI)	Complementary feeding practice AOR (95% CI)	Overall IYCF practice AOR (95% CI)
***Religion***			
Muslims	Ref.	–	–
Others	1.3 (0.7–2.2)		
***Age at first marriage (in years)***			
<18	Ref.	Ref.	–
18–24	1.3 (0.9–1.7)	0.7 (0.5–1.1)	
25–49	1.6 (0.5–4.9)	0.6 (0.1–2.9)	
***Age of the child (in months)***			
0–5	–	–	Ref.
6–8			0.1 (0.1–0.2)*
9–11			0.1 (0.1–0.3)*
12–23			0.6 (0.4–0.9)*
***Age of the child (in months)***			
0–5	Ref.	–	–
6–23	3.2 (2.4–4.5)*		
***Age of the child (in months)***			
6–8	–	Ref.	–
9–11		2.5 (0.9–6.7)	
12–23		5.6 (2.6–12.2)*	
***Gender of children***			
Male	–	Ref.	Ref.
Female		1.8 (1.2–3.0)*	1.4 (1.0–2.0)
***Place of delivery***			
Health facility	Ref.	–	Ref.
Home	1.7 (1.2–2.4)*		1.3 (0.9–1.8)
Others	2.1 (1.4–3.2)*		1.3 (0.9–2.1)
***Division***			
Dhaka	Ref.	Ref.	Ref.
Chattogram	0.9 (0.6–1.3)	0.6 (0.3–1.2)	0.9 (0.5–1.4)
Barishal	0.7 (0.4–1.2)	0.9 (0.4–2.0)	0.8 (0.4–1.6)
Khulna	0.8 (0.4–1.5)	2.0 (0.9–4.6)	1.5 (0.8–3.0)
Rajshahi	0.8 (0.4–1.3)	0.7 (0.3–1.5)	0.7 (0.4–1.4)
Rangpur	1.5 (0.8–2.7)	1.9 (0.9–4.0)	1.9 (1.1–3.4)*
Sylhet	1.7 (1.0–2.7)*	0.6 (0.3–1.1)	1.0 (0.6–1.6)
***Wealth quintile***			
Poorest	–	Ref.	–
Poor		1.6 (0.8–2.9)	
Middle		1.7 (0.9–3.1)	
Rich		2.0 (1.0–4.0)*	
Richest		3.8 (1.9–7.7)*	
***IYCF knowledge***			
Inappropriate	Ref.	–	Ref.
Appropriate	1.5 (1.1–2.1)*		1.4 (1.0–1.9)

*Note:* *p-Value < 0.05.

### Determinants of complementary feeding practices

Complementary feeding practices were significantly associated with child age, gender, and household wealth status. Compared to children aged 6–8 months, those aged 12–23 months (AOR: 5.6; 95% CI: 2.6–12.2) had greater odds of receiving appropriate complementary feeding. Female children were more likely than males to receive appropriate complementary feeding (AOR: 1.8; 95% CI: 1.2–3.0). Additionally, children from the richest households had significantly higher odds of meeting complementary feeding guidelines (AOR: 3.8; 95% CI: 1.9–7.7) (Table 5).

### Determinants of overall IYCF practices

Overall IYCF practices were significantly associated with child age, region, and maternal IYCF knowledge. Odds of appropriate IYCF practices decreased significantly with increasing child age. Compared to infants aged 0–5 months, the odds decreased by 90% for those aged 6–8 months (AOR: 0.1; 95% CI: 0.1–0.2) and remained lower through 12–23 months. Mothers residing in Rangpur Division had higher odds of appropriate IYCF practices (AOR: 1.9; 95% CI: 1.1–3.4), and those with good IYCF knowledge were more likely to practice appropriate IYCF (AOR: 1.4; 95% CI: 1.0–1.9) (Table 5).

## Discussion

This study assessed the levels and determinants of IYCF knowledge and practices among rural mothers in Bangladesh using nationally representative data from the BIHS 2018–2019. The findings offer a detailed overview of mothers’ awareness of recommended IYCF practices, their actual feeding behaviours, and the sociodemographic and contextual factors shaping these practices. Fewer than one-third of rural mothers demonstrated adequate IYCF knowledge, and a similar proportion adhered to recommended practices. Maternal education, household wealth, and geographic region emerged as significant predictors of IYCF knowledge, while adherence to recommended practices was strongly associated with the child’s age and geographic region.

Overall, maternal IYCF knowledge was relatively high for certain areas, such as the importance of EIBF (95.4%) and colostrum feeding (94.7%). In contrast, knowledge was markedly lower for topics such as the recommended frequency of breastfeeding (12.5%) and appropriate complementary feeding practices. These findings indicate that while some core components of IYCF have been effectively communicated to mothers, significant gaps remain in their comprehensive understanding of optimal feeding practices. This pattern aligns with evidence from other settings in South Asia and Sub-Saharan Africa, where awareness of early breastfeeding practices is generally high, yet more nuanced aspects of IYCF, such as feeding frequency and dietary diversity are often poorly understood.^(18,20,23,32,35,36)^

Regarding practices, although all children had been breastfed at some point, only 59% received breast milk within the first hour of birth. This rate is higher than that reported in BDHS-2022^(8)^ and MICS 2019,^(37)^ but remains below global targets. The higher estimates observed in this study may partly reflect differences in data sources and population coverage. The BIHS primarily represents rural households, whereas BDHS provides nationally representative estimates including both rural and urban populations.^(8,29)^ Variations in sociodemographic characteristics, health service utilisation, and cultural practices between these populations may contribute to the observed differences. Exclusive breastfeeding prevalence (70.3%) exceeded national estimates, indicating relatively better adherence in rural areas. However, complementary feeding practices were notably poor in our study, only 25.8% of children met the minimum acceptable diet, and dietary diversity was particularly low (30.1%). These estimates are lower than those reported in the BDHS-2022, which indicates gradual improvements in complementary feeding practices in recent years.^(8)^ The difference may be due to the time gap between datasets as well as differences in population coverage. Additionally, ongoing nutrition-specific and nutrition-sensitive interventions in Bangladesh may also have contributed to the improvements observed in the BDHS 2022. These findings underscore that while breastfeeding is widespread, the adequacy and diversity of complementary feeding remain suboptimal, a pattern also observed in many other low- and middle-income countries.^(38)^

Maternal education emerged as a strong predictor of IYCF knowledge, consistent with evidence from earlier studies.^(32,39,40)^ Higher education levels are often associated with improved cognitive abilities and critical thinking skills, which can facilitate the understanding and application of IYCF recommendations.^(41)^

In our study, mothers from higher wealth quintile households demonstrated significantly better IYCF knowledge compared to those from the poorest households. This may be attributed to greater access to information, educational opportunities, and health services among wealthier households, which can enhance awareness and understanding of optimal child feeding practices.

Mothers residing in Rangpur Division exhibited better IYCF knowledge, whereas those in Chattogram and Khulna Divisions demonstrated significantly lower knowledge levels. This finding should be interpreted with caution, as it may reflect characteristics specific to the rural households included in the BIHS rather than broader regional performance. In Bangladesh, divisions such as Dhaka, Khulna, and Chattogram generally show greater advancement in terms of higher education levels, better healthcare access, and greater maternal support services compared with other regions.^(42–44)^ Since this study utilised data from rural households, the observed differences may be influenced by localised programme coverage, survey sampling, or context-specific sociocultural factors, rather than reflecting long-term or nationwide regional trends.

Appropriate breastfeeding practices were more common among older infants and young children than in the youngest age group, possibly reflecting the continuation of breastfeeding beyond early infancy as recommended by WHO.^(1)^ In contrast, appropriate complementary feeding was markedly better among older children compared to those at the start of the complementary feeding period, which may be due to gradual dietary expansion, increased feeding frequency, and greater caregiver experience in providing diverse foods. However, overall IYCF practice was poorer among older age groups compared to the youngest, suggesting that while specific practices like continued breastfeeding or complementary feeding may improve with age, sustaining the full package of optimal feeding practices becomes challenging as children grow.

Our study found that children born in health facilities were less likely to receive appropriate breastfeeding. While this contrasts with some findings from Bangladesh,^(45)^ it is consistent with evidence from Pakistan, where facility-based deliveries were associated with lower rates of EIBF.^(46)^ This paradoxical association may be attributed to the higher prevalence of csarean deliveries or medical interventions in health facilities, which can delay the initiation of breastfeeding within the first hour after birth.

Consistent with previous research, this study found appropriate maternal IYCF knowledge was significantly associated with proper breastfeeding practices.^(47)^ Mothers with better IYCF knowledge are more aware of recommended feeding techniques and the benefits of timely and exclusive breastfeeding, which encourages proper breastfeeding practices.^(32)^

Complementary feeding practices were more prevalent among female children in our study. This is consistent with research from Belize, which reported higher rates of exclusive breastfeeding and dietary diversity among female children, highlighting potential gender-based differences in infant care.^(48)^ Such disparities may reflect cultural norms, household dynamics, and maternal education, where female children sometimes receive greater attention and resources. Notably, these findings represent a reversal of traditional trends in some settings,^(49)^ emphasising the need for further research to understand context-specific gender influences on complementary feeding.

In line with earlier studies, this study found that children from the richest households had significantly higher odds of meeting complementary feeding practices.^(50–52)^ This finding highlights the influence of household socioeconomic status on complementary feeding practices. Children from wealthier families are more likely to live in food-secure households, enabling caregivers to provide a diverse and adequate diet that meets recommended feeding standards.^(53)^

### Policy implications

Persistently low IYCF knowledge and practices among rural mothers in Bangladesh, together with influencing factors such as maternal education, household wealth, and geographic region, highlight the necessity for more targeted strategies. Existing IYCF programmes should be extended beyond caregiver knowledge transfer to address structural and social barriers that hinder behaviour change. Community-based approaches – such as nutrition education and awareness sessions involving fathers, grandmothers, and local health workers – can foster supportive environments for optimal feeding practices. Strengthening postnatal counselling within health systems, particularly for mothers from low-wealth households delivering in health facilities, is also critical to ensure timely initiation and continuation of breastfeeding and complementary feeding. Additionally, region-specific messaging and tailored interventions are needed to reduce geographic disparities in IYCF practices. Such approaches may include adapting nutrition education messages to local languages and cultural practices, addressing region-specific barriers to optimal feeding, and strengthening community-based counselling and health service delivery in underserved areas.

## Strengths and limitations

This study has several strengths, including the use of nationally representative rural data and robust multivariate analyses. It provides a comprehensive assessment of IYCF knowledge, breastfeeding practices, complementary feeding practices, and overall IYCF practices, along with their associated factors among rural mothers in Bangladesh. The findings are generalisable to rural populations in Bangladesh and may also be relevant to similar low-resource rural settings in South Asia. However, certain limitations should be noted. The use of multiple-response knowledge questions may have led to overestimation of maternal knowledge, and the absence of standardised scoring for knowledge and practices limits comparability with other studies. Additionally, the cross-sectional design restricts causal inferences.

## Conclusion

This study reveals substantial gaps in IYCF knowledge and practices among rural mothers in Bangladesh. While awareness of EIBF and colostrum feeding was high, deficiencies persisted in feeding frequency and practices during childhood illness. The study findings further highlighted that maternal education, geographic region, and household wealth were significant factors associated with IYCF knowledge, whereas child age and sex, place of delivery, geographic region, household wealth, and level of IYCF knowledge significantly influenced overall IYCF practices. The presence of multiple influencing factors suggests that knowledge alone may not be sufficient to ensure optimal feeding practices. Targeted, culturally tailored interventions addressing social and structural barriers are essential to translating knowledge into sustained optimal feeding practices and improving child nutrition outcomes.

## Supporting information

10.1017/jns.2026.10121.sm001 Sheikh et al. supplementary material Sheikh et al. supplementary material

## Data Availability

The data supporting this study have been deposited in the Harvard Dataverse (https://dataverse.harvard.edu/dataset.xhtml?persistentId=doi:10.7910/DVN/NXKLZJ).

## Supplementary material

The supplementary material for this article can be found at https://doi.org/10.1017/jns.2026.10121.

## Table 2. Long description

A table comparing the knowledge and practices of mothers regarding infant and young child feeding (IYCF). The table has 18 rows and 4 columns. The columns are labeled ‘Item no.’, ‘Knowledge of mothers regarding IYCF’, ‘Knows (%)’, and ‘IYCF indicators’ with a sub-column ‘Prevalence (%)’. Row 1: Item no., 1; Knowledge of mothers regarding IYCF, Time of initiation of breastfeeding after birth; Knows (%), 95.4; IYCF indicators, Ever breastfed (EvBF); Prevalence (%), 100. Row 2: Item no., 2; Knowledge of mothers regarding IYCF, Feeding colostrum; Knows (%), 94.7; IYCF indicators, Early initiation of breastfeeding (EIBF); Prevalence (%), 59. Row 3: Item no., 3; Knowledge of mothers regarding IYCF, Times of breastfeeding; Knows (%), 12.5; IYCF indicators, Exclusively breastfeeding under six months (EBF); Prevalence (%), 70.3. Row 4: Item no., 4; Knowledge of mothers regarding IYCF, What to do when a mother thinks that her baby is not getting enough milk; Knows (%), 78; IYCF indicators, Mixed milk feeding under six months (MixMF); Prevalence (%), 15.7. Row 5: Item no., 5; Knowledge of mothers regarding IYCF, Whether infants under six months should be fed water if the weather is very hot; Knows (%), 48; IYCF indicators, Continued breastfeeding 12–23 months (CBF); Prevalence (%), 93.7. Row 6: Item no., 6; Knowledge of mothers regarding IYCF, Whether breastfeeding infants under six months should be fed water if mothers are pregnant; Knows (%), 65.8; IYCF indicators, Introduction of solid, semi-solid or soft foods 6–8 months (ISSSF); Prevalence (%), 65.7. Row 7: Item no., 7; Knowledge of mothers regarding IYCF, Duration of exclusive breastfeeding; Knows (%), 90.4; IYCF indicators, Minimum dietary diversity 6–23 months (MDD); Prevalence (%), 30.1. Row 8: Item no., 8; Knowledge of mothers regarding IYCF, Age to start giving liquid (including water) other than breastmilk; Knows (%), 71.8; IYCF indicators, Minimum meal frequency 6–23 months (MMF); Prevalence (%), 64.8. Row 9: Item no., 9; Knowledge of mothers regarding IYCF, Age to start giving foods in addition to breastmilk; Knows (%), 69.1; IYCF indicators, Minimum meal feeding frequency for non-breastfed children 6–23 months (MMFF); Prevalence (%), 26.8. Row 10: Item no., 10; Knowledge of mothers regarding IYCF, Duration of breastfeeding; Knows (%), 67.2; IYCF indicators, Minimum acceptable diet 6–23 months (MAD); Prevalence (%), 25.8. Row 11: Item no., 11; Knowledge of mothers regarding IYCF, Consequences of iron deficiency in children; Knows (%), 85.3; IYCF indicators, Egg and/or flesh food consumption 6–23 months (EFF); Prevalence (%), 49.9. Row 12: Item no., 12; Knowledge of mothers regarding IYCF, Seasoning is often fortified with iodine; Knows (%), 55.6; IYCF indicators, Sweet beverage consumption 6–23 months (SwB); Prevalence (%), 10.1. Row 13: Item no., 13; Knowledge of mothers regarding IYCF, Duration of the need for extra meals for children after illness; Knows (%), 24.6; IYCF indicators, Unhealthy food consumption 6–23 months (UFC); Prevalence (%), 25.4. Row 14: Item no., 14; Knowledge of mothers regarding IYCF, What to do regarding child feeding when a child aged less than 6 months has diarrhea; Knows (%), 89.5; IYCF indicators, Zero vegetable or fruit consumption 6–23 months (ZVF); Prevalence (%), 44.8. Row 15: Item no., 15; Knowledge of mothers regarding IYCF, What to do regarding child feeding when a child aged more than 6 months has diarrhea; Knows (%), 96.3; IYCF indicators, Bottle feeding 0–23 months (BoF); Prevalence (%), 12.8. Row 16: Item no., 16; Knowledge of mothers regarding IYCF, When to wash hands; Knows (%), 100; IYCF indicators, No indicator specified; Prevalence (%), Not specified. Row 17: Item no., 17; Knowledge of mothers regarding IYCF, Ways to encourage young children to eat their food; Knows (%), 98.1; IYCF indicators, No indicator specified; Prevalence (%), Not specified. Row 18: Item no., 18; Knowledge of mothers regarding IYCF, Foods needed for a young child (<24 months) to grow and develop their brain; Knows (%), 99.8; IYCF indicators, No indicator specified; Prevalence (%), Not specified.

Navigate back to Table 2..
