# Supplementary material for: Infant and young child feeding in rural Bangladesh: insights into knowledge, practices, and associated factors
Source: J Nutr Sci. 2026 Jun 29;15:e50. doi: 10.1017/jns.2026.10121 (PMC13312372; doi:10.1017/jns.2026.10121)
Supplement: Sheikh et al. supplementary material [file S2048679026101219sup001.docx]

**Supplementary Table 1**. Unadjusted estimates of factors associated with IYCF knowledge

| **Variables** | **COR (95% CI)** | **P value** |
| --- | --- | --- |
| ***Education status of the mother*** | | |
| No education | Ref. |  |
| Primary incomplete | 1.14 (0.56–2.32) | 0.718 |
| Primary completed | 1.77 (0.93–3.39) | 0.083 |
| Secondary incomplete | 1.91 (1.06–3.44) | 0.030 |
| Secondary completed | 1.98 (0.98–4.00) | 0.057 |
| More than secondary | 3.94 (1.91–8.14) | < 0.001 |
| ***Occupational status of the mother*** | | |
| Housewife | Ref. |  |
| Others | 1.86 (1.06-3.25) | 0.031 |
| ***Religion*** | | |
| Muslims | Ref. |  |
| Others | 1.39 (0.85-2.23) | 0.196 |
| ***Age of the child (in months)*** | | |
| 0-5 | Ref. |  |
| 6-8 | 1.61 (1.02–2.58) | 0.043 |
| 9-11 | 1.08 (0.63–1.86) | 0.770 |
| 12-23 | 1.09 (0.76–1.56) | 0.641 |
| ***Division*** | | |
| Dhaka | Ref. |  |
| Chattogram | 0.78 (0.51-1.18) | 0.238 |
| Barishal | 1.40 (0.81-2.42) | 0.236 |
| Khulna | 0.34 (0.15-0.75) | 0.007 |
| Rajshahi | 0.75 (0.43-1.32) | 0.325 |
| Rangpur | 1.64 (0.98-2.75) | 0.062 |
| Sylhet | 0.66 (0.42-1.05) | 0.079 |
| ***Wealth quintile*** | | |
| Poorest | Ref. |  |
| Poor | 1.59 (1.04–2.44) | 0.034 |
| Middle | 1.41 (0.90–2.20) | 0.130 |
| Rich | 2.15 (1.34–3.44) | 0.001 |
| Richest | 3.07 (1.85–5.08) | < 0.001 |

**Supplementary Table 2**. Unadjusted estimates of factors associated with breastfeeding practice

| **Variables** | **COR (95% CI)** | **p value** |
| --- | --- | --- |
| ***Religion*** | | |
| Muslims | Ref. |  |
| Others | 1.35 (0.81–2.25) | 0.250 |
| ***Age at first marriage (in years)*** | | |
| <18 | Ref. |  |
| 18-24 | 1.27 (0.96–1.68) | 0.097 |
| 25-49 | 1.83 (0.65–5.17) | 0.254 |
| ***Age of the child (in months)*** | | |
| 0-5 | Ref. |  |
| 6-23 | 3.00 (2.20–4.09) | < 0.001 |
| ***Place of delivery*** | | |
| Health facility | Ref. |  |
| Home | 1.78 (1.30–2.43) | < 0.001 |
| Others | 1.75 (1.19–2.59) | 0.005 |
| ***Division*** | | |
| Dhaka | Ref. |  |
| Chattogram | 0.84 (0.57–1.24) | 0.380 |
| Barishal | 0.83 (0.48–1.42) | 0.492 |
| Khulna | 0.62 (0.35–1.10) | 0.102 |
| Rajshahi | 0.71 (0.43–1.17) | 0.180 |
| Rangpur | 1.47 (0.85–2.56) | 0.171 |
| Sylhet | 1.66 (1.06–2.59) | 0.026 |
| ***IYCF knowledge*** | | |
| Inappropriate | Ref. |  |
| Appropriate | 1.50 (1.10–2.04) | 0.011 |

**Supplementary Table 3**. Unadjusted estimates of factors associated with complementary feeding practice

| **Variables** | **COR (95% CI)** | **p value** |
| --- | --- | --- |
| ***Age at first marriage (in years)*** | | |
| <18 | Ref. |  |
| 18-24 | 0.67 (0.45–0.99) | 0.047 |
| 25-49 | 0.54 (0.12–2.48) | 0.431 |
| ***Age of the child (in months)*** | | |
| 6-8 | Ref. |  |
| 9-11 | 1.98 (0.76–5.13) | 0.162 |
| 12-23 | 5.08 (2.40–10.73) | < 0.001 |
| ***Gender of children*** | | |
| Male | Ref. |  |
| Female | 1.58 (1.07–2.33) | 0.021 |
| ***Division*** | | |
| Dhaka | Ref. |  |
| Chattogram | 0.65 (0.37–1.14) | 0.130 |
| Barishal | 0.69 (0.31–1.52) | 0.356 |
| Khulna | 1.41 (0.66–3.00) | 0.372 |
| Rajshahi | 0.72 (0.35–1.51) | 0.387 |
| Rangpur | 1.25 (0.64–2.44) | 0.507 |
| Sylhet | 0.60 (0.32–1.12) | 0.109 |
| ***Wealth quintile*** | | |
| Poorest | Ref. |  |
| Poor | 1.56 (0.87–2.80) | 0.132 |
| Middle | 1.56 (0.87–2.80) | 0.132 |
| Rich | 1.77 (0.96–3.27) | 0.070 |
| Richest | 2.83 (1.49–5.41) | 0.002 |

**Supplementary Table 4**. Unadjusted estimates of factors associated with overall IYCF practice

| **Variables** | **COR (95% CI)** | **p value** |
| --- | --- | --- |
| ***Age of the child (in months)*** | | |
| 0-5 | Ref. |  |
| 6-8 | 0.11 (0.06–0.23) | < 0.001 |
| 9-11 | 0.14 (0.07–0.29) | < 0.001 |
| 12-23 | 0.63 (0.46–0.88) | 0.006 |
| ***Gender of children*** | | |
| Male | Ref. |  |
| Female | 1.29 (0.97–1.72) | 0.085 |
| ***Place of delivery*** | | |
| Health facility | Ref. |  |
| Home | 1.31 (0.95–1.81) | 0.099 |
| Others | 1.27 (0.85–1.89) | 0.239 |
| ***Division*** | | |
| Dhaka | Ref. |  |
| Chattogram | 0.77 (0.51–1.18) | 0.235 |
| Barishal | 0.75 (0.41–1.37) | 0.343 |
| Khulna | 1.18 (0.64–2.15) | 0.597 |
| Rajshahi | 0.75 (0.42–1.32) | 0.311 |
| Rangpur | 1.55 (0.92–2.62) | 0.097 |
| Sylhet | 1.02 (0.66–1.57) | 0.937 |
| ***IYCF knowledge*** | | |
| Inappropriate | Ref. |  |
| Appropriate | 1.26 (0.92–1.72) | 0.151 |

**Supplementary Table 5.** Assumptions check for regression model (Factors associated with IYCF knowledge)

| **Factors** | **Collinearity statistics** | | **Hosmer and Lemeshow test** | **Nagelkerke R square** | **Omnibus test** |
| --- | --- | --- | --- | --- | --- |
|  | **Tolerance** | **VIF** |  |  |  |
| Education of mother | 0.79 | 1.25 | 0.674 | 0.105 | 0.000 |
| Occupation of mother | 0.98 | 1.01 |  |  |  |
| Age of child | 0.99 | 1.00 |  |  |  |
| Religion | 0.97 | 1.02 |  |  |  |
| Division | 0.96 | 1.04 |  |  |  |
| Wealth quintile | 0.78 | 1.28 |  |  |  |

**Supplementary Table 6.** Assumptions check for regression model (Factors associated with overall IYCF practice)

| **Factors** | **Collinearity statistics** | | **Hosmer and Lemeshow test** | **Nagelkerke R Square** | **Omnibus test** |
| --- | --- | --- | --- | --- | --- |
|  | **Tolerance** | **VIF** |  |  |  |
| Age of child | 0.99 | 1.00 | 0.935 | 0.160 | < 0.001 |
| Place of delivery | 0.98 | 1.00 |  |  |  |
| Gender of child | 0.95 | 1.05 |  |  |  |
| Division | 0.94 | 1.05 |  |  |  |
| IYCF knowledge | 0.99 | 1.00 |  |  |  |
